# Pulsatility hemodynamics during speed modulation of continuous‐flow total artificial heart in a chronic in vivo model

**DOI:** 10.1111/aor.14237

**Published:** 2022-03-31

**Authors:** Taiyo Kuroda, Takuma Miyamoto, Chihiro Miyagi, Anthony R. Polakowski, Christine R. Flick, Barry D. Kuban, George B. Voros, Kimberly Such, Kiyotaka Fukamachi, Jamshid H. Karimov

**Affiliations:** ^1^ Department of Biomedical Engineering Cleveland Clinic Lerner Research Institute Cleveland Ohio USA; ^2^ Biological Resources Unit Cleveland Clinic Cleveland Ohio USA; ^3^ Cleveland Clinic Lerner College of Medicine of Case Western Reserve University Cleveland Ohio USA

**Keywords:** heart failure, mechanical circulatory support, pulsatile flow, pulsatility index, total artificial heart

## Abstract

**Background:**

The evaluation of pulsatile flow created by the new Cleveland Clinic continuous‐flow total artificial heart (CFTAH100), which has a re‐designed right impeller and motor, had not been tested in vivo. The purpose of this study was to evaluate the feasibility of pulsatility with the CFTAH100 during the application of pump speed modulation protocols in a chronic animal model.

**Methods:**

A 30‐day chronic animal experiment was conducted with a calf. Five pulsatile studies were performed on the alert animal. The mean pump speed was set at 2800 rpm, and modulated sinusoidally within a range of 0 to ± 35% of mean speed, in increments of 5% at 80 beats per minute (bpm). The pressures and pump flow were collected and a pulsatility index (PI) was calculated.

**Results:**

The calf was supported with the CFTAH100 without any major complications. The maximum and minimum pump flows changed significantly from baseline in all conditions, while the mean pump flow did not change. All flow pulsatility (FP) readings in all conditions significantly increased from baseline, and the percent modulation (%S) and FP had a strong positive correlation (*r* = 0.99, *p*  <  0.01). The PI also increased significantly in all conditions (maximum at %S of 35%, 2.2  ±  0.05, *p* < 0.01), and a positive correlation between %S and PI (*r* = 0.99, *p* < 0.01) was observed.

**Conclusion:**

The CFTAH100 showed the feasibility of creating pulsatile circulation with sinusoidal pump speed modulation.

## Graphical abstract

The pulsatile circulation created with the Cleveland Clinic Total Artificial Heart was evaluated in a 30‐day chronic in vivo experiment. The pump demonstrated the feasibility of creating pulsatile circulation.

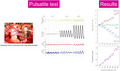

## 1 BACKGROUND

Peripheral vascular dysfunction has been reported among patients supported by long‐term, pulse‐diminished, continuous‐flow left ventricular assist devices (LVADs).^1^, ^2^ The endothelial disorder may be associated with this peripheral vascular dysfunction and may lead to adverse cardiovascular events after LVAD implantation.^3^ Hence, the feasibility of generating pulsatility will be an important evaluation item for mechanical circulatory support devices.

The Cleveland Clinic continuous‐flow total artificial heart (CFTAH) is a self‐regulating centrifugal pump with a single rotating assembly (rotor) supported by a hydrodynamic bearing. The rotor has an impeller on each end and is capable of moving freely in the axial direction, influenced by the pressure differences of the left and right chambers, which balance the right and left sides of the heart.^4^ We previously reported the 90‐day survival of calves with our CFTAH080, which demonstrated reliable hemodynamic output.^5^ The CFTAH080 also demonstrated the feasibility of creating pulsatility with speed modulation.^6^, ^7^

Recently, to improve biocompatibility, a revised device, the CFTAH100, was created. The CFTAH100 has a new motor designed to increase the axial magnetic force and a redesigned right impeller.^8^, ^9^ The increase of the axial magnetic force enables the rotor to stay within the appropriate positional range to avoid contact with the housing walls of either chamber; however, the ability to create pulsatility with the CFTAH100 had to be demonstrated. Therefore, we designed an experiment to evaluate the feasibility of pulsatility with the CFTAH100 on an alert calf. Also, to explore more detailed effects of pulsatility, we designed the experiment to have a more finely divided speed modulation application. The purpose of this study was to evaluate the feasibility of pulsatility with our new CFTAH100 during the application of speed modulation protocols in a long‐term chronic animal model.

## 2 METHODS

### 2.1 Pump description

**FIGURE 1:**
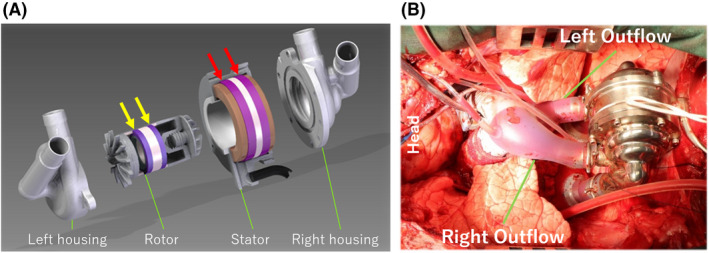
(A) Three‐dimensional exploded view. Yellow arrows show the stacked motor with two magnet rings in the rotor. Orange arrows show the two steel laminations in the stator. These are separated by a non‐magnetic spacer (in white). (B) Cleveland Clinic continuous‐flow total artificial heart implanted in the chest

The Cleveland Clinic CFTAH (Figure 1A) consists of four parts: left and right pump housings, rotor, and stator assembly. The magnetic forces allow a limited window of free axial movement in response to pressure differences between the right and left filling chambers. This axial movement shifts the right impeller's distance from the aperture, which influences the right pump output and allows passive differential pressure regulation of the atrial chambers. Excessive axial motion is limited by magnetic restoring forces created by the magnetic design of the motor.

Nevertheless, there are extreme situations that exceed the normal axial restoring force, such as left or right inlet suction, or other extreme conditions as experienced during implant surgery. In our previous version (the CFTAH080), a plastic insert was bonded in the left housing to prevent scratches on the left housing from impeller contact in the event of left suction at high pump speed. The improved axial restoring force would eliminate the need for the insert, prompting the design of a new motor with a stacked spacing of magnets and laminations. Our novel stacked motor has two magnet rings in the rotor and two steel laminations separated by a non‐magnetic spacer, with increased axial restoring force at the edges of the positional range, and less restoring force at the center of this range.

**FIGURE 2:**
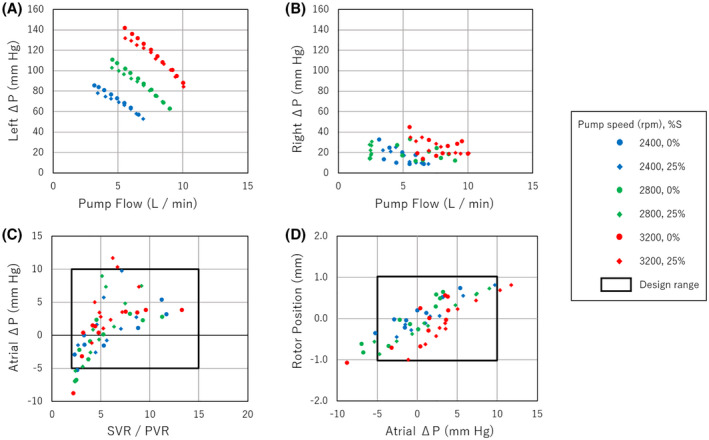
(A) The left pump head curve of the CFTAH100. (B) The right pump head curve of the CFTAH100. (C) The left/right balance among different conditions. (D) The rotor position among different conditions. Atrial Δ*P*, atrial delta pressure; left Δ*P*, left delta pressure; PVR, pulmonary vascular resistance; right Δ*P*, right delta pressure; SVR, systemic vascular resistance

Figure 2A,B show the in vitro head curve relationships of the CFTAH100 in pressure rise versus pump flow. Both left and right pumps were operated in the designed range, with and without ± 25% sinusoidal speed modulation, which generated the pulsatile flow. The pressure rise (or delta pressure) of the left pump was calculated by the difference between the aortic pressure and left atrial pressure. Also, the pressure rise (or delta pressure) of the right pump was calculated by the difference between the pulmonary arterial pressure and the right atrial pressure. The plots in the right pump head curve were scattered because the geometry of the right chamber changes by the pressure difference between the left and right inlets. Nevertheless, the head curve relationships were similar between non‐pulsatile flow and pulsatile flow. The left/right atrial balance is shown in the relationship with the ratio of systemic vascular resistance and pulmonary vascular resistance (Figure 2C). Figure 2D shows the relationships between rotor position and atrial delta pressure. The newly designed motor kept the rotor in the design range.

### 2.2 Anesthesia and surgical techniques

The study was approved by Cleveland Clinic's Institutional Animal Care and Use Committee, and the animals received humane care in compliance with the “Guide for the Care and Use of Laboratory Animals” (Institute of Laboratory Animal Resources, Commission on Life Sciences, National Research Council, National Academy Press, Washington, DC, 2011) and institutional guidelines.

We conducted a 30‐day chronic animal experiment using a 3‐month‐old male Jersey calf. The CFTAH100 was implanted in the calf (88.0 kg at implant) under general anesthesia and a median sternotomy surgery (Figure 1B). The procedure was performed using our usual method with cardiopulmonary bypass, as described in the previous publication.^5^ CFTAH inlet cuffs and outlet grafts were sutured to the annulus of the tricuspid valve and the mitral valve for the inlet, pulmonary artery, and aorta for the outlet. The fluid‐filled pressure catheter line was also implanted in the left carotid artery to obtain the systemic pressure as the aortic pressure (AoP). Also, the fluid‐filled pressure lines for the right atrial pressure (RAP) and left atrial pressure (LAP) were connected to the branch located at the inflow cuffs, and pulmonary artery pressure (PAP) was placed through the pressure port located at the outflow graft. Placed pressure lines were exteriorized through the left chest.

After the surgery, the animal was transferred to the chronic care unit and continuously monitored during the study. Pump parameters, pressure data, and the animal's general condition, including vital signs and appetite, were recorded during the entire study. Also, plasma‐free hemoglobin and lactate dehydrogenase (LDH) were collected on POD 1, 3, 7, 10, 14, 21, and 28 to analyze the hemocompatibility of the CFTAH100. The animal was extubated on POD 0, and the autopsy was performed after 30 days.

### 2.3 Pulsatility testing protocol

**FIGURE 3:**
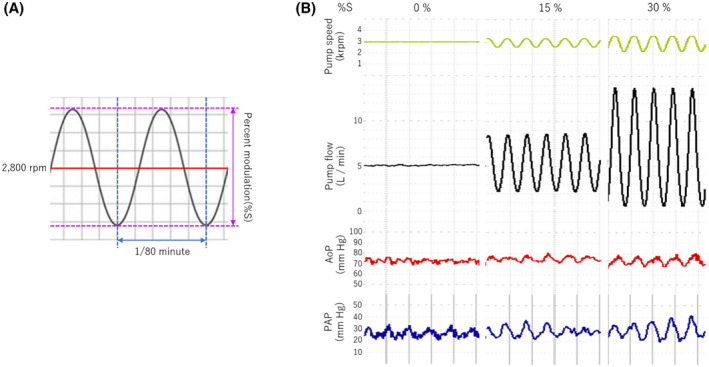
(A) Depiction of a generated sinusoidal wave. (B) Actual waveforms of pump speed, pump flow, aortic pressure (AoP), and pulmonary artery pressure (PAP) with percent modulation (%S) of 0%, 15%, and 30%

We performed five pulsatility studies (POD 13, 16, 17, 21, and 23) on an awake calf. To generate the pulsatility, CFTAH100 pump speed was set to a mean speed of 2800 rpm, and the speed was modulated sinusoidally within different ranges of ± 0%, ± 5%, ± 10%, ± 15%, ± 20%, ± 25%, ± 30%, and ± 35% (percent modulation: %S) (Figure 3A). The modulation frequency was 80 beats per minute. The RAP, LAP, PAP, AoP, pump flow, and power consumption of the pump were collected during the speed modulation (Figure 3B). The pulse pressure of the AoP and PAP were calculated by subtracting minimum pressure from maximum pressure in a modulation cycle. The pump flow was calculated using the pump current and pump power, which was validated with in vitro bench testing that duplicated the case. Flow pulsatility (FP) was calculated by subtracting minimum pump flow from maximum pump flow in a flow cycle. Finally, the pulsatility index (PI) was calculated using the formulas PI = FP/mean pump flow.

### 2.4 Data collection and statistical analysis

The data were processed using the software LabChart pro, ver. 8.1.9 (ADInstruments, Colorado Springs, Colorado, USA) and recorded at 200 Hz. The values were expressed as mean ± standard deviation. Their differences were assessed by a one‐way analysis of variance. For significant interactions, the Bonferroni multiple comparisons were performed. A *p*‐value of less than 0.05 was considered statistically significant. The correlations were assessed using Spearman's correlation coefficients. Statistical analyses were processed using the software EZR on R commander ver. 1.40 (Y Kanda, Tochigi, Japan).

## 3 RESULTS

**FIGURE 4:**
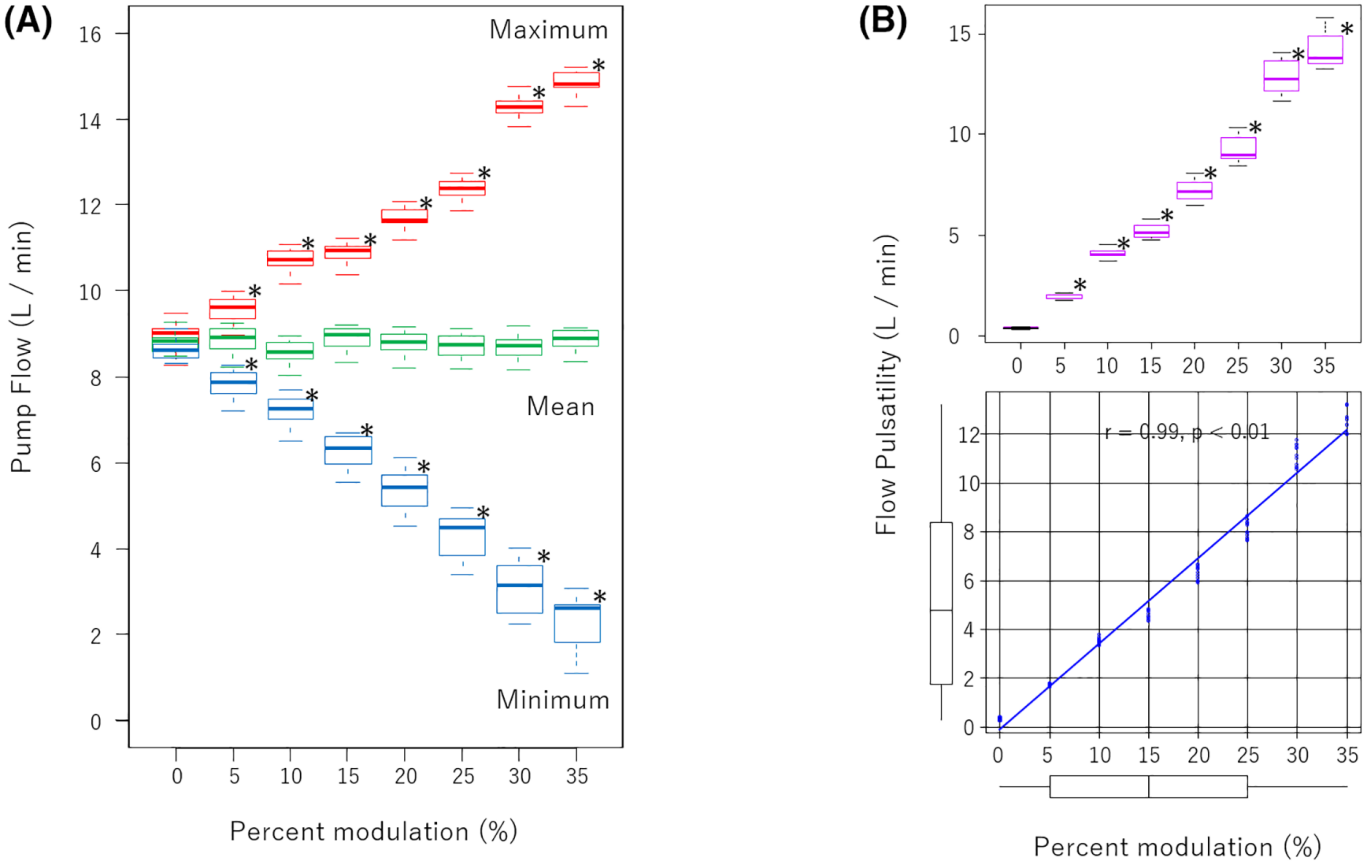
(A) Pump flow with different percent modulation (%S). (B) top: Flow pulsatility versus %S. bottom: Correlation between flow pulsatility and %S analyzed with Spearman's rank correlation coefficient. *p < 0.01 compared with 0% of %S

The maximum pump flow was 8.9 ± 0.3 L/min at a constant speed of 2800 rpm (%S of 0%), which falls within the intended performance range of the CFTAH. It increased to 9.6 ± 0.3 L/min (*p* < 0.01) when the %S was set to 5%. When the %S comes to 10%, which is the setting used most often during this 30‐day study, maximum pump flow was recorded as 10.7 ± 0.3 L/min. This flow amount was an increase of approximately 1 L/min from the baseline. The maximum pump flow continued to increase linearly, with an increase of %S up to 35% (*p* < 0.01). The actual values of the maximum pump flow were 10.9 ± 0.3, 11.7 ± 0.3, 12.4 ± 0.3, 14.3 ± 0.3, and 14.8 ± 0.3 L/min at %S 15%, 20%, 25%, 30%, and 35%, respectively (Figure 4A). According to these results, the maximum pump flow at an %S of 35% recorded was approximately 5 L/min increase from the baseline.

The pump minimum flow was 8.8 ± 0.3 L/min at a constant speed of 2800 rpm (%S of 0%), and it was also the intended performance range of the CFTAH. It decreased to 7.8 ± 0.2 L/min (*p* < 0.01) by %S to 5%. As with the maximum pump flow, the minimum pump flow continued to decrease linearly with an increase of %S up to 35% (*p* < 0.01). The actual values of the minimum pump flow were 7.2 ± 0.4, 6.2 ± 0.4, 5.4 ± 0.5, 4.3 ± 0.6, 3.1 ± 0.7, and 2.3 ± 0.7 L/min at %S of 10%, 15%, 20%, 25%, 30%, and 35%, respectively. The minimum pump flow was approximately 5.2 L/min less than baseline; however, the pump remained stable even though the CFTAH's hydrodynamic bearing is not designed for low speeds.

The mean pump flow was 8.8 ± 0.3 L/min at baseline, which did not change significantly in all conditions. It indicated that the total amount of provided pump flow did not increase or decrease despite the maximum and minimum pump flow change. FP was 0.3 ± 0.04 L/min at baseline, and it increased to 1.7 ± 0.1 L/min at %S of 5%. At %S of 10%, FP was observed as 3.5 ± 0.1 L/min. FP continued to increase linearly up to %S of 35% (*p* < 0.01). The observed flows are 4.6 ± 0.2, 6.3 ± 0.3, 8.1 ± 0.3, 11.1 ± 0.4, and 12.6 ± 0.5 L/min at %S of 15%, 20%, 25%, 30%, and 35%, respectively. Therefore, FP increased approximately 1.75 L/min per 5% increase in speed modulation depth. In addition, %S and FP had a strong positive correlation statistically (*r* = 0.99, *p* < 0.01) (Figure 4B).

**FIGURE 5:**
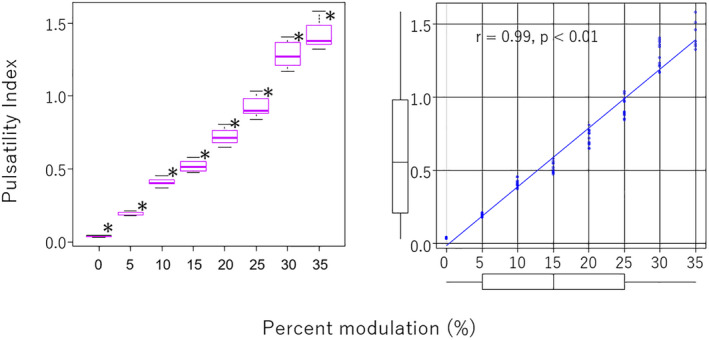
Left: Pulsatility index versus percent modulation (%S). Right: Correlation between flow pulsatility and %S analyzed with Spearman's rank correlation coefficient. *p < 0.01 compared with 0% of %S

Regarding the PI, the more we increased %S, the higher the PI went (Figure 5). At a %S of 5%, the PI was calculated as 0.2 ± 0.004; when we set the %S to 10%, the PI increased to 0.4 ± 0.03. The PI showed a continuous linear increase at all conditions of %S (*p* < 0.01) as follows: the PI was 0.5 ± 0.04 at %S 15%, 0.7 ± 0.05 at %S 20%, 0.9 ± 0.07 at %S 25%, 1.3 ± 0.09 at %S 30%, and 1.4 ± 0.1 at %S 35%. This data also demonstrated a positive correlation between %S and PI (*r* = 0.99, *p* < 0.01).

**FIGURE 6:**
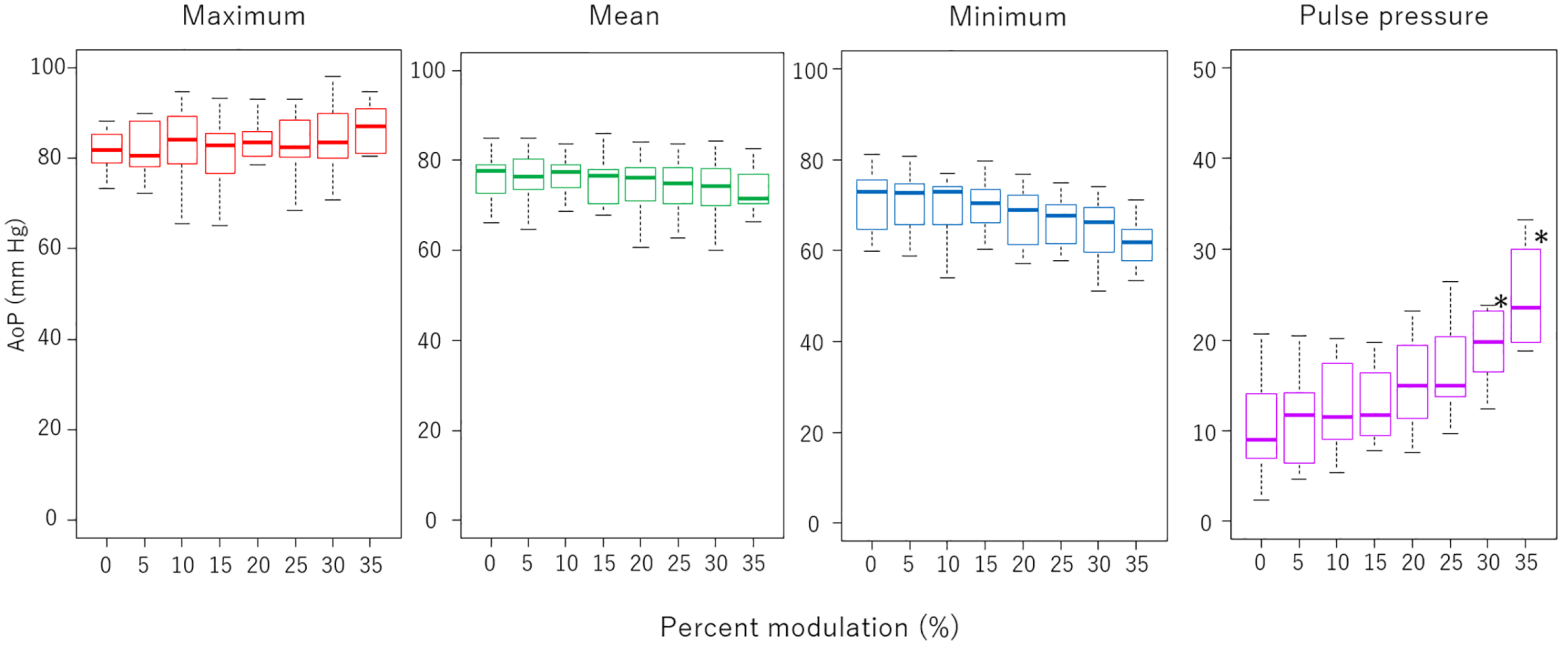
Aortic pressure (AoP) and aortic pulse pressure in different percent modulations (%S). *p < 0.01 compared with 0% of %S

**FIGURE 7:**
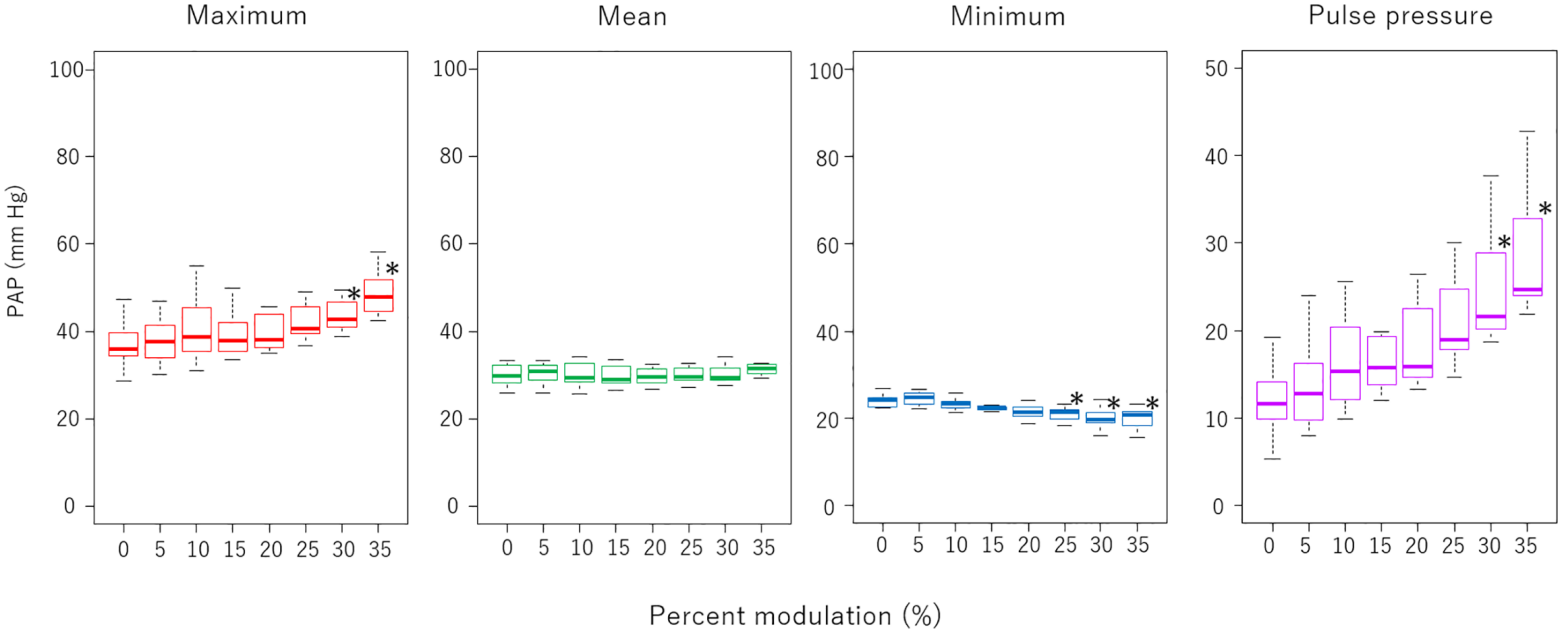
Pulmonary artery pressure (PAP) and pulmonary artery pulse pressure in different percent modulations (%S). *p < 0.01 compared with 0% of %S

Regarding the obtained pressures, a pulse pressure trended higher as the %S increased; however, only the values at 30% (20.7 ± 6.4 mm Hg) and 35% (25.0 ± 6.2 mm Hg) were significantly higher (*p* < 0.01) (Figure 6). The maximum AoP, mean AoP, and minimum AoP did not show statistically significant differences. As for other pressures, there was a decrease in minimum PAP at an %S of 25% (21.0 ± 1.5 mm Hg, *p* = 0.01), 30% (20.0 ± 2.2 mm Hg, *p* < 0.01), and 35% (19.9 ± 2.8 mm Hg, *p* < 0.01) (Figure 7). Also, significant increases in maximum PAP at %S of 30% (44.5 ± 5.0 mm Hg, *p* = 0.04) and 35% (48.8 ± 5.6 mm Hg, *p* < 0.01), and in pulse pressure of PAP at 30% (24.5 ± 6.2 mm Hg, *p* < 0.01) and 35% (29.0 ± 7.6 mm Hg, *p* < 0.01), were observed. We did not find any significant difference in LAP or RAP, including pulse pressure. Moreover, the pressure difference between LAP and RAP is the major factor that moves the rotor in the axial direction; however, the data showed no significant difference at any speed modulation.

For hemocompatibility, the plasma‐free hemoglobin was 4.9 mg/dl at baseline (before implantation), and the level peaked at POD 7 (11.6 mg/dl) and then went down to normal level (2.0–4.7 mg/dl), but then went up at POD 28 (25.9 mg/dl). However, LDH did not show a spike in the trend and remained at almost the same level (1684–1768 U/L) during the study. In addition, LDH was 1187 U/L at the baseline and peaked at POD 1 (2652 U/L).

During the duration of the experiment, the animal had no symptoms and had a stable general condition, including vital signs, appetite, sleep, and physical activities. The postoperative averages of the mean AoP and PAP were 94.8 ± 14.4 and 34.2 ± 3.1 mm Hg, respectively. Also, the mean LAP and RAP were 15.5 ± 3.3 and 12.8 ± 2.2 mm Hg, respectively. The design limits of atrial pressure difference are between −5 and 10 mm Hg for systemic‐to‐pulmonary vascular resistance (SVR / PVR) ≥ 2.0. This was achieved, with the exceptions of POD 0 and 1 (LAP = 20.2 mm Hg, RAP = 13.2 mm Hg, LAP‐RAP = 7.1 mm Hg), and POD 23 (LAP = 11.0 mm Hg, RAP = 18.3 mm Hg, LAP–RAP = −7 mm Hg). Therefore, the calf was supported for 30 days post‐implant without any complications. The calf weight was 95.7 kg (+ 7.7 kg from the implant) on POD 21 and 100.0 kg before euthanasia (+ 12.0 kg from the implant).

At the planned autopsy, no signs of organ enlargement, hemorrhage, or congestion were found. No gross thromboemboli were detected in any of the internal organs. The slice of the lung showed small, dark‐colored old thrombi inside the bronchi. The brain specimen inspection did not show any thrombus. The post‐explant pump inspection revealed no visible signs of mechanical wear on the surfaces of the left housing or the outboard faces of the impeller blades. There were some expected minor thrombus formations at the junction of the pump ports and silicone graft of both the outflow and inflow assemblies. The thrombus formations were affixed to the titanium pump ports.

## 4 DISCUSSION

In this study, the FP increased significantly with the increase in %S. Also, the analysis described a strong correlation between %S and FP. These data demonstrate that the Cleveland Clinic CFTAH100, which has a new design of the motor and right impeller, generates pulsatility with speed modulation of the rotor. As for the pump flow, the mean pump flow did not significantly change, although the maximum and minimum pump flows did. This shows that the CFTAH100 is creating precise sign wave flow. In addition, the PAP was highly pulsatile because of the CFTAH‐specific action of the right aperture during the speed modulation. The impact of this phenomenon is unknown; however, it did not result in major lung bleeding or congestion, since we did not find evidence in the autopsy.

To evaluate the effectiveness of the pulsatility, we also calculated the PI for this study and found that the CFTAH100 increased PI with speed modulation. Witman, et al. stated in their report that there is a significant positive relationship between PI and peripheral vascular function evaluated by flow‐mediated vasodilation (FMD).^1^ FMD is a value that has been used for the assessment of endothelial function.^10^, ^11^ According to their report, increased PI with speed modulation of the CFTAH100 may cause a positive effect on peripheral vascular function.

Furthermore, Shimamura, et al. reported the linear positive relationship between PI and ocular blood flows in goats with an implanted continuous‐flow LVAD.^12^ Also, according to this report, pulsatile circulation with a higher PI would have better peripheral circulation. On the contrary, Cortese, et al. reported that the continuous‐flow LVAD was not related to worsening endothelial function, comparing FMD in the patient between LVAD, hypertension, and heart failure.^13^ Thus, the effect of the pulsatile flow on the endothelial function is still controversial; however, we believe that the CFTAH100 has at least shown the potential to create higher PI circulation support.

Regarding the axial restoring force of the new motor, data described the well‐balanced condition of the rotor during the pulsatile study. The difference between LAP and RAP was not significantly changed in the application of %S change, indicating that the self‐regulating capability is unaffected by speed modulation. This suggested that the greater axial force to move the rotor, which pushes the rotor toward the housing, had not been generated during the pulsatile study. Also, the pump included a Hall‐effect sensor to track the axial rotor position. Unfortunately, we could not get data from this sensor because it began malfunctioning on POD 12; however, no abnormalities in the rotor position were observed while it was functional. Additionally, we did not observe any scratch marks in the left housing or the top of the impeller blades when we disassembled the pump. Therefore, contact between the housing and impeller blades had not occurred, which leads us to conclude that the new motor was well‐balanced between the left and right ventricular pressures.

The autopsy revealed no gross thromboemboli in the internal organs. Small, dark‐colored thrombi were observed in the bronchi; however, it seemed very old and was explainable by the intra‐tracheal tube used during the surgery.

There are some limitations in this early report. The study was performed on one animal. However, the data collection was successfully recorded in multiple data points. The data collection process was repeated at five planned data points during the experiment, and the protocol was *repeated* and subsequently three times during the pulsatility data collection process as defined by the study protocol. A total of 86 data points were collected for this extensive analysis. The data showed a significant difference. Nevertheless, the CFTAH100 functioned as intended and similar to the in vitro data. In addition, we believe the testing protocol, activity of the animal, and complex interaction of atrial balancing feature with pump performance rendered this study unique. Also, the pump flow measurement was successfully estimated from a calculation using current. This value is adjusted with the data from in vitro bench testing, which duplicated the case.

## 5 CONCLUSIONS

The Cleveland Clinic CFTAH with a new stacked motor design has demonstrated the generation of near‐physiologic pulsatile flow through speed modulation. The generated pulsatile flow also demonstrated a strong positive correlation with the pulsatility index.

## CONFLICT OF INTEREST

Barry D. Kuban, Kiyotaka Fukamachi, and Jamshid H. Karimov are co‐inventors of the device. Other co‐authors have no disclosures.

## AUTHOR CONTRIBUTIONS

Taiyo Kuroda: Data collection, data analysis, manuscript drafting, and preparation. Takuma Miyamoto, Barry D. Kuban, George B. Voros, and Kimberly Such: Data collection, data analysis, manuscript preparation. Chihiro Miyagi, Anthony R. Polakowski, and Christine R. Flick: Data collection, data analysis, critical manuscript review. Kiyotaka Fukamachi: Critical article revision, data analysis. Jamshid H. Karimov: Study design and approval, data analysis.
